# The National Association of Dental Examiners

**Published:** 1897-11

**Authors:** 


ARTICLE VI.

### THE NATIONAL ASSOCIATION OF DENTAL EXAMINERS.

EDITORIAL IN *Dental Practitioner and Advertiser.*

In the last number of this journal we gave our impression of what would be the consequences of the persistence of the National Board of Dental Examiners in the course which they seemed to have marked out for themselves. It is a great deal of satisfaction when disaster overtakes us, or seems imminent, to be able to say, "I told you so," however ungracious and irritating it may prove. We warned the members of the Examiners Association that their aggressive and unwarranted action would be resisted by the colleges, and would not tend to further the objects which they desired. There are members of the Association of Faculties who are as earnest in their labors for the advancement of the educational standard as can be any member of the Examiners Association. They also know how that can be brought about a great deal better than does any one outside.

All of these men were but too well aware that the co-operation of the Examining Boards, if not essential to success, would certainly aid in securing it. They knew that the colleges which were not so earnestly in favor of an advance stood quite ready to join issue on the question, and that they only sought for a plausible excuse to resist the onward movement. Any attempt at coercion would afford that very pretext. The result has justified predictions, and the aggressive action of the Examiners Association resulted in a retrogressive instead of progressive movement in the Faculties Association. Again we should like to repeat the warning, that for the Examiners to go out of their province and assume to dictate to the colleges will be to invite defeat, and to retard progress indefinitely. The colleges cannot be thus driven. There will certainly be defensive action taken. If the Examiners attempt to carry out their threat of placing upon a black list and branding as unrecognized any institutions, it matters not how weak they may be, so long as they are recognized by their own State laws and the National Association of Faculties, the United States Courts will give them quick and effectual relief, and will punish such an assumption of power on the part of unauthorized persons. On this point the highest legal authorities are agreed.

We spoke plainly in the last number, because we thought that the occasion demanded plain words. We had a perfect right to examine into the qualifications of the members of the various Boards for the self-assumed task of placing an authoritative stamp of approval or disapproval upon our schools, and of demanding that they should conform to an arbitrary standard which unauthorized persons had erected. We still think that we were right. But we wish to disclaim any personal animus, or to be understood as calling into question any individual or professional status or standing. Some of those who have made themselves conspicuous in the invasive attitude of the Examiners Association, have expressed the feeling that we were personal in some of our expressions. We feel certain that anyone who knows us as we really are, will not make this charge. The men whom we most freely criticise are usually our dearest friends. Why! If we can not call a friend to account; in Heaven's name whom can we bring to book? We can discuss a principle, and show wherein we believe that our best loved associate is wrong, without for a moment questioning his moral qualities.

We can learn nothing by antagonizing our enemies, if we have any. It is only those who are nearest to us who can do us good by taking the opposite side. If there is any one for whose good opinion and personal esteem we do not care, it is very certain we shall not step outside our path to endeavor to show him wherein we believe him to be wrong.

In the editorial article published in the July number, occur some expressions which subsequent knowledge has convinced us were founded upon misinformation. This we deeply regret, for though we may be very earnest, we intend to be just. What was said was upon the supposition that our source of information was reliable. But the assertion that one of the men who obtained the incorporation of the National Association of Examiners held an unearned diploma, and that their action was self-assumed, we are convinced was an error, and all inferences which may have been drawn from those supposed facts are and were unwarranted.

But this does not change what we believe to be the truth, that the National Association of Dental Examiners is overstepping its rights and privileges when it attempts to force the colleges to acknowledge its paramount authority, and to accept unquestioned its dicta. While individual members are amply qualified for such an office, if it were permissible, as a whole that body is incompetent.

In the first place, the Examiners Association has no standard for admission to its ranks. If through political chicanery or influence the most incompetent man in the whole profession is appointed to a State Examining Board, he is received by them without question. There is no provision that he shall have any special professional knowledge whatever, or that he shall be acquainted with the college curricula. It is not even necessary that he should be a dentist. He may be the most notorious quack imaginable, but if he has in any way obtained an appointment to a Board, he straightway becomes a judge in matters professional and ethical. The Examiners Association should establish some standard of its own, before it assumes to dictate one for other professional bodies.

It is undisputedly charged that less than half the members of the State Examining Boards are graduates from colleges. How can men sit in judgment upon matters of which they are ignorant? With what justice can they pronounce upon the qualifications of men in affairs of which they know nothing? They may be good practitioners and eminent men, but they must know what is and what should be the course of study pursued in our colleges before they can properly sit in judgment upon that course.

They therefore exceed their just functions when they assume jurisdiction over the colleges and their courses of study. They are supposed to be appointed for other purposes. There is plenty for them to do in looking to the enforcement of the various laws. Illegal practitioners abound in almost every State. There is a loud call for the suppression of irregular practice. Only the State Boards can properly look after these matters. And yet the actual enforcement of the laws on the statute books is almost a farce. Let the Boards look to these legitimate duties if they would serve their profession.

It is claimed that some of the colleges have graduated illiterate and unfit men. That is undoubtedly true. Yet in the Faculties Association there is a constant effort being made to remedy this, and year by year the standard is being raised Only for the mischievous interference of the Examiners the ground gained last year could have been maintained. As it is, under the provocation of the unwise action of the Examiners, considerable has been lost. Those struggling for better things found that the Philistines were upon them when their locks were shorn. They were bound hand and foot, their eyes were put out, and sport was made of them in the temples of their opponents. The representatives on whose aid they had relied were in the toils of some seductive Delilah, the lust for office or the hope of gain. The standard already secured was placed upon the sacrificial altar, and its friends were powerless to rescue it, because the action of the Examiners had maddened its enemies. The friends of education need the co operation instead of the opposition of the Examiners, until their locks are again grown, when they will pull down the temple of the Philistines, if they cannot be freed. Better all perish together than drag out an ignoble existence.

That some graduates have not the preliminary qualifications which they should have is of course true. One member of the Examiners Association, in a seeming desire still further to embarrass those who are earnestly striving for better things, publishes a list of absurd answers given before State Examining Boards. That is a sword that cuts both ways. Members of the Faculties Association have lists of questions exhibiting quite as great ignorance on the part of Examiners. The most absurd miscomprehensions of scientific facts are set forth in these questions, which seem called from books that the Examiners did not understand. A description of arteries and nerves that have no existence has been demanded. Terms have been used in many cases that are unknown to science. Positive contradictions of known facts are involved in queries. Lists of these are in existence quite as astounding as anything that ean be culled from the answers of students, and the temptation to publish them is great, but the knowledge that all these things only embarrass those who are working for a better state of affairs, and will embitter a controversy that should not exist at all, prevent the retort which might be made to an unwise and injudicious member of a State Examining Board.

The Examiners Association persisted in a course against which the best friends of dental education protested. Some colleges may perhaps be intimidated into compliance with these demands. But there are those which, while they establish even higher requirements than anything that the Examiners Association demands, will not submit to insulting dictation. They desire co-operation and assistance in raising the standard, but they will not sign away their self-respect, and become the vassals of those whom they cannot admit as their masters in knowledge, or their superiors in integrity and honor.

---

ARTICLE VII.

### THE NATIONAL ASSOCIATION OF DENTAL FACULTIES.

